# Photoscopy (Skiascopy or Retinoscopy)

**Published:** 1907-02

**Authors:** 


**Photoscopy** (Skiascopy or Retinoscopy). By **Mark D. Stevenson,** M.D., Ophthalmic Surgeon to the Akron City Hospital; Oculist to the Children's Home, Akron, Ohio. Octavo of 126 pages, illustrated. Philadelphia and London: W. B. Saunders Company, 1906. (Cloth, $1.25 net).

"Photoscopy is the method of estimating the refractive error of an eye, by reflecting light into it by a plane or concave mirror, and observing the size, shape, brilliancy, direction, and rate of movement of an area (image) of light apparently in the observed eye."

We have thought of no better way to introduce a notice of this book than to quote the author's definition of its subject-title, as given in his opening paragraph. It at once discloses his purpose—namely, to describe the most approved methods of practising photoscopy.

While there are already several excellent works describing the test variously called retinoscopy, skiascopy and shadow-test, Dr. Stevenson has, under the term photoscopy, given us one of the most clearly written and best manuals on this subject. For reasons which he gives he thinks this name to be more accurate than any of the others yet proposed. The work begins, as we have already remarked, with a definition of photoscopy, emphasises its value, and describes the necessary instruments and their use. The author outlines the optical principles underlying the test, and by illustrative experiments and observations he shows how they are applied to the determination of the refractive condition of the eye. In this method of examination he prefers the use of the plane to the concave mirror, in the great majority of cases. The book contains numerous original illustrations and is well made up. A historical sketch and a splendid bibliography follow. We cordially recommend the work as a trustworthy guide to this subject and congratulate the author on the success which he has attained in presenting the topic.

